# Let the Body Talk: Preliminary Data of an Open Trial of Dance Movement Therapy for Eating Disorders

**DOI:** 10.3390/jcm13010005

**Published:** 2023-12-19

**Authors:** Ilaria Bastoni, Anna Guerrini Usubini, Maria Gobetti, Mila Sanna, Glenda Pagnoncelli, Laura Uboldi, Valentina Villa, Gianluca Castelnuovo, Alessandro Sartorio, Leonardo Mendolicchio

**Affiliations:** 1 Psychology Research Laboratory, Istituto Auxologico Italiano, Istituto di Ricovero e Cura a Carattere Scientifico (IRCCS), 20145 Milan, Italy; i.bastoni@auxologico.it (I.B.); v.villa@auxologico.it (V.V.); gianluca.castelnuovo@auxologico.it (G.C.); 2 Centro Arti Terapie, 20122 Milan, Italy; milasanna.studio@gmail.com (M.S.); glendapagnoncelli@gmail.com (G.P.); laurauboldipsi@gmail.com (L.U.); 3 Department of Psychology, Catholic University of Milan, 20123 Milan, Italy; maria.gobetti01@icatt.it; 4 Politecnico di Milano—METID: Metodi e Tecnologie Innovative per la Didattica, 20133 Milan, Italy; 5 Experimental Laboratory for Auxo-Endocrinological Research, Istituto Auxologico Italiano, Istituto di Ricovero e Cura a Carattere Scientifico (IRCCS), 20145 Milan, Italy; sartorio@auxologico.it; 6 Division of Eating and Nutrition Disorders, Istituto Auxologico Italiano, Istituto di Ricovero e Cura a Carattere Scientifico (IRCCS), 28824 Verbania, Italy; l.mendolicchio@auxologico.it; 7 Experimental Laboratory for Metabolic Neurosciences, Istituto Auxologico Italiano, Istituto di Ricovero e Cura a Carattere Scientifico (IRCCS), 28824 Verbania, Italy

**Keywords:** dance movement therapy, eating disorders, emotional regulation, alexithymia, interoceptive awareness

## Abstract

Background: There is growing support for considering Dance Movement Therapy (DMT) as an effective approach to improving physical and psychological symptoms in eating disorders (ED), but additional evidence is needed. The current study aims to investigate the effectiveness of a DMT intervention for inpatients with ED during an in-hospital rehabilitation program for ED in reducing emotion dysregulation and alexithymia and improving interoceptive awareness. Methods: Forty-nine consecutive inpatient young women with ED (aged between 18 and 34 years) recruited from a clinical center for the rehabilitation of obesity and ED received four group sessions of DMT intervention. All participants completed the Difficulties in Emotion Regulation Scale (DERS), the Toronto Alexithymia Scale (TAS), and the Multidimensional Assessment of Interoceptive Awareness Scale (MAIA) before (Time 0) and after the intervention (Time 1). Paired-sample t-tests were run to assess differences between Time 0 to Time 1. Results: From pre-to-post interventions, there was a significant reduction in the means of all of the subscales of DERS, suggesting an improvement in emotion regulation competencies, with the only exception for difficulties in awareness that increased (*p* = 0.016). We also found a significant reduction in alexithymia, as proved by significant differences in all of the subscales and the total score of TAS (*p* < 0.001), and significant improvements in interoceptive awareness as suggested by increased scores of the noticing (*p* = 0.043), emotional awareness (*p* < 0.001), body listening (*p* < 0.001), and trusting (*p* < 0.001) subscales of MAIA. Conclusion: Overall, our results point towards the efficacy of dance/movement in reducing symptoms of eating disorders. Our findings also suggest that dancing can be considered a useful intervention to increase emotional regulation, reduce alexithymia, and enhance interoceptive awareness.

## 1. Introduction

Among the spectrum of weight-related disorders, the prevalence of eating disorders (ED) such as anorexia nervosa (AN), bulimia nervosa (BN), and binge-eating disorder (BED) is increasing worldwide, especially in conjunction with of the COVID-19 pandemic [1,2,3]. Several studies pointed out that during the COVID-19 pandemic, the incidence of ED increased in addition to a worsening of their symptomatology, due to the restrictive measures imposed by governments to deal with the pandemic spread [4]. Specifically, Taquet and colleagues found that in 2020, the incidence of ED was 15.3% higher than in the previous years, in a sample of almost 5.2 million people. In addition, the incidence of ED primarily concerned young females with anorexia nervosa, and it was also associated with suicidal ideation and suicide attempts [5]. Termorshuizen and colleagues found that during the COVID-19 pandemic, restrictive behaviors of people with anorexia nervosa and binge episodes of people with bulimia nervosa and binge-eating disorders (BED) dramatically increased [6]. Similar findings were replicated in Italy, where the rates of ED increased markedly during the COVID-19 pandemic [7].

The etiology of ED is complex and research has found several underlying factors such as biological, psychological, and environmental ones [8]. Among others, many theories have been proposed that suggest that dysfunctional eating behaviors are involved as coping strategies to deal with emotions supporting the hypothesis of a crucial role of emotion regulation in eating behavior patterns [9,10,11].

Emotion regulation is a multidimensional construct described by Gratz and Roemer [12] as the ability to understand, be aware, accept, and modulate responses to emotions, and act in accordance with personal values and desires instead of emotional cues. Several studies have pointed out that people with ED show a deficit in functionally regulating emotions. On the contrary, they generally involve maladaptive coping strategies such as avoidance to manage emotions [13].

The inability to functionally manage one’s own emotions is related to alexithymia [13]. Alexithymia has been defined as the inability to recognize and describe one’s own emotions and differentiate between feelings and bodily sensations [14]. Despite alexithymia being present in several psychiatric conditions, such as depression [15], schizophrenia [16], and post-traumatic stress disorder [17], it was found to be relevant in people with ED. In this regard, several studies have reported higher levels of alexithymia in people with ED compared to controls, suggesting that people with ED have specific difficulties in identifying and communicating their emotions [18].

Beyond emotion regulation difficulties, a deficit in interoceptive awareness was also found to play a key role in the development and maintenance of ED [19]. Interoceptive awareness can be defined as the ability to recognize bodily sensations and it is related to emotion regulation [20]. Several studies suggest that while adequate interoceptive awareness is crucial for regulating food intake, on the contrary, poor interoceptive awareness was related to increased disordered eating [21]. Together these findings suggest that the core of eating-related psychopathology is represented by emotional, interpersonal, and interoceptive difficulties that should be the target of treatment for ED [22].

Most of the evidence-based clinical guidelines for ED recommend psychological interventions as a core component of the treatment that is based on nutritional, psychological, and physical therapy. Cognitive-behavioral therapy is considered the first-line therapy for ED, followed by family-based interventions, particularly suited for younger patients. Other evidence suggests psychodynamic and interpersonal psychotherapy as effective approaches in treating ED [23]. Despite the effectiveness of gold-standard treatments having been demonstrated, evidence for their long-term results seems to be inconsistent to date [20].

Body image disturbances are one of the core elements in ED [24,25] with several implications at the cognitive, emotional, and behavioral levels. It has commonly been reported that patients with ED have negative body experiences and difficulties in bodily awareness. Such difficulties need to be targeted in treatments effectively [26].

Dance Movement Therapy (DMT) is a therapeutic approach, born in the 1940s, centered on the body, and based on the use of expressive movement for improving physical and psychological health. Among art therapies, DMT is focused on the use of bodily movement in a therapeutic way, to encourage the awareness of thoughts and emotions that cannot be differently expressed [26]. Arising from the fundamental connection between body and mind, DMT promotes the expression of internal contents through movement, in order to help individuals to express themselves and their internal states, without using words. DMT emerged in contrast to standard psychotherapies, such as cognitive behavioral therapy which emphasizes talking as the prevalent form of communication. Rather, DMT focuses on the human body as the primary way of communication and expression [27].

Although many forms of DMT are various and different from each other, DMT is generally delivered in groups and emphasizes the relationship between therapist and patient(s).

DMT has been proposed as a treatment for improving health-related psychological outcomes and well-being in several physical and psychological illnesses such as breast cancer, dementia [28], fibromyalgia [29], autism spectrum disorder [30], brain trauma [31], hypertension [32], Parkinson’s disease [33], and depression [34].

Considering ED, only one pilot study conducted by Savidaki and colleagues [26] explored the effects of a 14-week-long DMT intervention for people with ED. The results of this work showed clinically and statistically significant improvements in body image from pre-to-post intervention. In another study, a DMT intervention was administered in women with obesity, showing significant decreases in psychological distress and body image distress, as well as decreased emotional eating and improved self-esteem. Although the sample did not include people with ED, the study is relevant because it showed that DMT impacted emotional eating, psychological distress, and body image, which are all factors related to ED [35]. These results provide evidence about the potential effectiveness of DMT in treating ED, but further confirmations are necessary.

Thus, the aim of the current study is to investigate the effects of a brief DMT intervention in patients with ED within the context of a multidisciplinary inpatient rehabilitation program for ED. Specifically, we expected to find improvements in emotion dysregulation, alexithymia, and interoceptive awareness as a result of the intervention we proposed.

As mentioned before, there is initial evidence in the literature of the suitability of DMT for Eds; however, only a pilot study has evaluated the effectiveness of a DMT intervention on body image and alexithymia in patients with ED [26]. Unfortunately, the intervention was not replicable in our context, because of its length (fourteen weeks) which is incompatible with the length of the recovery (four weeks) when the present study would take place and did not target our outcomes of interest. For this reason, we developed an ad-hoc DMT intervention to be administered within the context of a rehabilitation program for weight management for patients with EDs and we preliminarily assessed its efficacy.

## 2. Materials and Methods

### 2.1. Participants and Procedures

The participants were forty-nine young women aged between 18 and 34 years with ED (34 = AN; 8 = BN; 7 = BED) consecutively recruited from the IRCCS Istituto Auxologico Italiano–Piancavallo (VB) a third-level clinical center for the rehabilitation of obesity and ED, located in the north of Italy. Participants were selected for the study if they were female (given the higher prevalence of eating disorders in females than males [36], aged between 18 and 35 years, and were diagnosed with ED, according to the Diagnostic and Statistical Manual of Mental Disorders, Fifth Edition (Structured Clinical Interview–Clinical Version 5 (SCID-5 CV))). All of the patients provided written and informed consent to participate. Participants were excluded if they had a comorbid psychiatric disorder diagnosed following the criteria of the DSM-5 or any other medical condition that could compromise their participation in the study. Participants included in the study were at their first access at our third-level center, with a previous history of only outpatient visits.

At the beginning of their rehabilitation program, patients who met the inclusion/exclusion criteria were recruited with a clinical interview conducted by a clinical psychologist, after being informed about the research and asked to complete written informed consent to participate. The Structured Clinical Interview for DSM-5 (SCID-5) was used as a screening tool for psychiatric diagnoses. Once enrolled, participants were asked to complete self-report questionnaires to collect demographical and clinical variables of interest for the study. After completing the pre-intervention assessment (Time 0), the participants received the DMT intervention which was delivered as part of the rehabilitation program during the four weeks of rehabilitation. At the end of the intervention, just before discharge from the hospital, all participants were asked to complete the same post-intervention assessment (Time1).

The study was approved by the Ethical Committee of the Istituto Auxologico Italiano (code of identification: 03C921_2019). All procedures were run following the Helsinki Declaration of 1975 and its later amendments or comparable ethical standards.

The schedule of enrolment, assessment, and intervention is presented in Table 1.

### 2.2. Measures

Clinical data were collected via self-report using Italian-validated and widely used questionnaires. Body mass index (BMI: kg/m^2^) was carefully measured to the nearest 0.1 kg with an electronic scale at the beginning of the program, according to the internal procedure of the hospital.

Emotion regulation. The Difficulties in Emotion Regulation Scale (DERS) [12] is a self-report questionnaire consisting of 36 items, rated on a 5-point Likert scale ranging from 1 (almost never) to 5 (almost always), which explores the following subscales and a total score: non-acceptance, goals, impulse, awareness, strategies, and clarity, which respectively concern non-acceptance of negative emotions, inability to undertake purposeful behavior when experiencing negative emotions, difficulty in controlling impulsive behavior when experiencing negative emotions, lack of awareness of one’s emotions, limited access to effective emotion regulation strategies, lack of understanding of the nature of one’s emotional responses. Higher scores indicate higher difficulties in emotion regulation. We used the Italian version validated by Giromini and colleagues [37]. In our sample, the Cronbach alphas of non-acceptance, goals, impulse, awareness, strategies, and clarity were 0.890, 0.865, 0.876, 0.787, 0.909, and 0.875, respectively.

Alexithymia. The Toronto Alexithymia Scale (TAS) [38] is a self-report questionnaire consisting of 20 items rated on a 5-point Likert scale ranging from 1 (strongly disagree) to 5 (strongly agree). We used the Italian validated version [39]. The TAS-20 is composed of three factors: difficulty in modulating and identifying feelings (Factor 1); difficulty in describing one’s feelings to others (Factor 2); externally oriented thinking (Factor 3). In our sample, the Cronbach alphas of Factor 1, Factor 2, and Factor 3 were 0.852, 0.656, and 0.623.

Interoceptive awareness. The Multidimensional Assessment of Interoceptive Awareness scale (MAIA; Ref. [40] is a self-report questionnaire consisting of 32 items rated on a 6-point Likert scale ranging from 0 (never) to 5 (always) which explores the following subscales: noticing (all of the body sensations), not-distracting (from discomfort or pain), not-worrying (when sensations of discomfort or pain are experienced), attention regulation (to regulate attention to body sensations), emotional awareness (to connect body sensations to underlying emotional states), self-regulation (the ability to manage distress by listening to body sensations), body listening (listening to the body sensations), and trusting (experiencing one’s body). We used the Italian version [41]. In our sample, the Cronbach alphas of noticing, not-distracting, not-worrying, attention regulation, emotional awareness, self-regulation, body listening, and trusting were 0.608, 0.344, 0.639, 0.851, 0.880, 0.826, 0.863, and 0.757, respectively.

### 2.3. Intervention

#### 2.3.1. The Multidisciplinary Rehabilitation Program for EDs

The hospital where the study was conducted offers a multidisciplinary and tailored inpatient treatment program for weight management with medical, dietitian, physical, and psychological components which were followed by all of the participants of the study. During the four weeks of hospitalization, patients are screened and placed on a tailored nutritionally balanced diet after a consultation with a dietician aiming to assess their nutritional status and evaluate adequate nutrient intake. Then, they received individual and group counseling provided by a dietitian, aimed at improving changes in eating habits. Furthermore, patients followed a physical activity program delivered by physiotherapists and trainers composed of postural exercises, walking, and physical strengthening. Psychotherapy was also provided. It was based on the gold-standard cognitive behavioral therapy for ED [12,36,42], and it was delivered once a week by a psychotherapist, both in individual and group settings, according to the individual’s needs.

#### 2.3.2. The DMT Intervention

Participants of the DMT group followed a DMT intervention within the context of the multidisciplinary rehabilitation program for weight management. It was delivered in consecutive groups composed of 9–10 participants each (5 groups in total) and it was carried out by a psychologist specialized in DMT. Sessions took place in a spacious gym of the hospital, where participants could move freely and easily use some tools and objects (paper, pens, balls). The intervention entailed 4 sessions, provided once a week, lasting about one hour each. Sessions are aimed at increasing interoceptive awareness in order to become able to recognize bodily sensations and improving emotion regulation which means becoming able to recognize, name, and regulate emotional experiences.

In accordance with the suggestions of Schmais and White [43], each session of our intervention was divided into four parts: the first was the initial phase in which participants were introduced to the DMT, prepared for the main activities of the therapy, and invited to express their current feelings, in order to begin to create a climate of sharing; in the second phase, participants were guided into the core of the therapy intervention in which the therapist presented and realized the main activities of the session; in the third step of the session, participants were accompanied to come into contact with their bodies and their deep thoughts and feelings, in order to reduce stress and increase their body self-awareness. In the final stage of the session, participants were given the opportunity to reflect on the experience and express reflections on the process. A detailed description of the sessions is presented in Appendix A.

### 2.4. Statistical Analysis

Descriptive statistics were conducted to investigate the baseline characteristics of the sample, including frequencies and percentages for categorical variables and means and standard deviations for continuous variables. Skewness and kurtosis indices were calculated to assess the normal distribution of the variables.

To assess differences from pre-to-post-DMT intervention in all of the variables of interest (DERS, TAS, MAIA), paired-sample t-tests were run. Cohen’s *d* effect size was used to quantify the magnitude of the reported effects.

Analyses were performed using Jamovi (The jamovi project 2021) (Version 1.6) [Computer Software]. Retrieved from https://www.jamovi.org.

### 2.5. Treatment Fidelity

The research team was composed of psychologists, psychotherapists, researchers, and doctoral students with expertise in the field of psychological interventions and research in healthcare settings.

In line with previous studies [38], treatment fidelity was assured with direct observation of at least 20% of the intervention sessions by two independent trained bachelor-level observers. Both of them carefully completed a checklist with all contents and exercises planned for each session, in order to monitor adherence to the protocol. Coders had to achieve a minimum of 80% reliability with each other. With a lower level of agreement, sessions were invalidated.

## 3. Results

### 3.1. Baseline Characteristics of the Sample

The baseline characteristics of the sample are shown in Table 2. The sample was composed of 49 young adult patients with ED aged between 18 and 34 years. The mean age was 23.1 (SD = 4.09), and the mean BMI was 19.1 (SD = 8.40). All of the variables were normally distributed.

### 3.2. Preliminary Evidence of Treatment Efficacy

A series of paired sample *t*-tests were run to assess differences in emotion regulation, alexithymia, and interoceptive awareness, by addressing changes in the means of the subscales and total scores of DERS, TAS, and MAIA, respectively, from Time 0 to Time 1.

As far as emotion regulation is concerned, the results showed a significant reduction in the means of all of the subscales of DERS, suggesting an improvement in emotion regulation competencies, with the only exception for the awareness subscale which increased from Time 0 to Time 1 suggesting a greater lack of emotional awareness (*p* = 0.016).

As far as alexithymia is concerned, the paired sample t-test revealed a significant reduction in all of the means of TAS from Time 0 to Time 1 suggesting that difficulties in modulating and identifying feelings (Factor 1; *p* < 0.001), difficulties in describing one’s feelings to others (Factor 2; *p* = 0.006), and difficulties in externally oriented thinking (Factor 3; *p* = 0.037) decreased from Time 0 to Time 1.

Finally, as far as interoceptive awareness is concerned, the results showed significant differences in the means of the noticing (*p* = 0.043), emotional awareness (*p* < 0.001), body listening (*p* < 0.001), and trusting (*p* < 0.001) subscales, which significantly increased from Time 0 to Time 1. No other statistically significant differences in MAIA subscales were found.

The results are presented in Table 3.

## 4. Discussion

The aim of the current study was to provide preliminary evidence of the effects of a brief DMT intervention in patients with ED within the context of a multidisciplinary inpatient rehabilitation program for ED.

Our preliminary analyses found a positive effect of our brief DMT intervention on emotional regulation, alexithymia, and interoceptive awareness in a sample of 49 young adult female patients with ED seeking a multidisciplinary rehabilitation program. In particular, we found a significant improvement in all of the subscales of DERS from Time 0 to Time 1 suggesting a decrease in difficulties in emotional regulation. However, the improvement of the awareness subscale indicates that difficulties in emotional awareness increased. Such a result was unexpected and counterintuitive. In addition, it is in contrast with the results that emerged by the administration of MAIA that comprised a similar subscale, the emotional awareness subscale, that improved from Time 0 to Time 1 (discussed below). This evidence leads us to hypothesize that the apparent increase in difficulties in the awareness subscale of DERS could reflect its poor performance as a subscale rather than a real effect of the intervention, as previously suggested by several studies.

Our study also found that alexithymia (TAS) decreased in all its dimensions, that is, difficulties in modulating and identifying feelings, difficulties in describing one’s feelings to others, and difficulties in externally oriented thinking. Similar findings were previously detected by Savidaki and colleagues [26] in their pilot study addressing the efficacy of DMT on body image and alexithymia in eating disorders. They found that alexithymia decreased from pre-to-post-DMT intervention; however, this change was not statistically significant. The authors discussed that the result was probably influenced by the small sample size and required future replications.

Finally, our analyses also showed a positive effect of DMT in interoceptive awareness. From Time 0 to Time 1, the noticing, emotional awareness, body listening, and trusting subscales of MAIA increased significantly, supporting that interoceptive awareness is a core element in DMT that could be improved with a therapeutic intervention [44]. Such a hypothesis is sustained in the literature. In the study of Millman and colleagues [45] evaluating two DMT tasks to reduce bodily detachment in patients with depersonalization-derealization disorder (DDD) compared with a healthy control group, the authors found that DMT tasks produced a significant reduction in symptoms and enhanced mindfulness and awareness of body sensations in patients with DDD. The authors also found that mean interoceptive awareness did not significantly improve after performing the tasks, but reduced overall DD symptom severity was still associated with elevated interoceptive awareness, suggesting a role for interoceptive processing in the attenuation of DD symptoms.

Our preliminary results seem to suggest that the DMT intervention was effective in reducing emotional regulation and alexithymia and improving interoceptive awareness in patients with ED. However, the results of the present study must be interpreted with caution due to several intrinsic limitations.. In the first instance, the small sample size and the scarce possibility of generalizing the results linked to the high specificity of the context where the study took place. Future replications of the study with a larger sample size will be mandatory to confirm these preliminary observations. Furthermore, our intervention was very short (only 4 weeks, i.e., related to the duration of our hospitalization), thus potentially being able to compromise the results. Lastly, the lack of a comparison with a control group, preventing us from separating the effectiveness of the DMT more in depth from the other treatments entailed in the multidisciplinary rehabilitation program.

In conclusion, our preliminary results seem to suggest that a short-term DMT intervention can exert positive effects on a sample of young adult female patients with ED seeking a multidisciplinary rehabilitation program for their clinical condition. Nevertheless, taking into consideration the above limitations of the study, future studies with a larger study group (compared with a control group without DMT) and conducted for more prolonged periods are necessary to confirm these promising results.

## Figures and Tables

**Table 1:** The study procedure.

	Pre-Intervention Time 0		Intervention	Post-Intervention Time 1
	Week 1	Week 1	Week 1-2-3-4	Week 4
Eligibility screen	X			
Informed consent	X			
Allocation		X		
Data collection: DERS TAS MAIA		X		
Intervention: Multidisciplinary rehabilitation program + DMT			X	
Data collection: DERS TAS MAIA				X

**Table 2:** Descriptive statistics of the sample.

Variable	N	Mean ± SD (Time 0)	Mean ± SD (Time 1)
Age (in years)	49	23.1 ± 4.09	
BMI (Kg/m^2^)	49	19.1 ± 8.40	
DERS			
Non-accept	49	19.6 ± 6.30	17.5 ±6.57
Goals	49	18.2 ± 3.31	16.8 ± 2.97
Impulse	49	17.4 ± 5.25	15.9 ± 4.95
Awareness	49	16.5 ± 4.80	18.1 ± 5.41
Strategies	49	27.3 ± 6.74	24.4 ± 6.54
Clarity	49	14.5 ± 2.69	13.4 ± 2.38
Tot	49	113.6 ± 19.53	106.2 ± 17.81
TAS			
Factor 1	49	25.8 ± 6.24	22.0 ± 6.79
Factor 2	49	18.3 ± 3.96	16.8 ± 4.81
Factor 3	49	18.8 ± 5.07	17.5 ± 4.97
Tot		62.9 ± 11.40	56.4 ± 12.88
MAIA			
Noticing	49	2.87 ± 1.03	3.16 ±1.03
Not-distracting	49	2.56 ± 0.98	2.32 ± 0.86
Not-worrying	49	2.045 ± 1.10	2.20 ± 1.30
Attention regulation	49	1.87 ± 1.04	2.06 ± 1.12
Emotional awareness	49	2.72 ± 1.24	3.41 ± 0.97
Self-regulation	49	1.50 ± 1.14	1.76 ± 1.34
Body listening	49	1.23 ± 1.01	1.90 ± 1.32
Trusting	49	0.86 ± 0.91	1.36 ± 1.39

Note: SD: Standard Deviation; BMI: Body Mass Index; DERS: Difficulties in Emotion Regulation scale; TAS: Toronto Alexithymia Scale; MAIA: Multidimensional Assessment of Interoceptive Awareness scale.

**Table 3:** Paired sample *t*-test.

Variables	Student’s t	*p*	Cohen’s d Effect Size
DERS			
Non-accept	2.47	0.017	0.353
Goals	3.14	0.003	0.449
Impulse	2.55	0.014	0.364
Awareness	−2.51	0.016	−0.359
Strategies	3.69	<0.001	0.527
Clarity	2.80	0.007	0.400
DERS TOT	3.51	<0.001	0.502
TAS			
Factor 1	4.92	<0.001	0.703
Factor 2	2.88	0.006	0.412
Factor 3	2.15	0.037	0.307
TAS tot	4.69	<0.001	0.670
MAIA			
Noticing	−2.079	0.043	−0.297
Not-distracting	1.528	0.133	0.218
Not-worrying	−0.740	0.463	−0.106
Attention regulation	−1.319	0.193	−0.188
Emotional awareness	−3.945	<0.001	−0.564
Self-regulation	−1.593	0.118	−0.228
Body listening	−0.4335	<0.001	−0.619
Trusting	−3.602	<0.001	−0.515

Note: DERS TOT: Difficulties in Emotion Regulation scale-tatal score; TAS: Toronto Alexithymia Scale; MAIA: Multidimensional Assessment of Interoceptive Awareness scale.

## Data Availability

Data will be available upon a reasonable request to the author A.G.U.

## Appendix A

**Table A1:** Description of therapeutic sessions of DMT.

Session Number	Domains	Activities
1	Emotion regulation and alexithymia	“Discovering yourself through the other” In this exercise, participants are in pairs. One participant is placed in front of another and both are asked to observe each other. The first participant is invited to think about a color and an emotion associated with it. Then, she has to express that emotion through a movement. The other member of the couple should repeat the same movement, being allowed to express the same or another emotion elicited by the movement she repeated.
2	Interoceptive awareness and alexithymia	“Be in a bubble” At the beginning of this exercise, participants are invited to find a comfortable position sitting or kneeling (not standing) and are asked to draw a circle around themself symbolically defining their space. With music, they have to imagine being inside a bubble inside that circle that gradually gets bigger and allows them to explore larger spatial dimensions. It is possible to carry it around, or even be transported by the bubble. Then, the bubble gets smaller and smaller until it forces the person to return to a fetal position. Finally, they can get out of the bubble with the help of someone outside of it, and then walk within the space. At the end of the exercise, participants are invited to express how they feel and discuss in the group their own experiences.
3	Interoceptive awareness	“We are our body” On a sheet of paper, participants are invited to make a portrait of themselves with a representation of their feelings at the moment within the body. They have to choose a sign of their emotions and try to move to symbolize them. During the exercise, participants are guided to breathe slowly and deeply reflect on their experience.
4	Interoceptive awareness and alexithymia	“Blind dance” In pairs, participants were blinded and placed one in front of another. Then, one starts to touch the other member of the couple in order to explore its body. Then, both try to make something together, like a movement. It is not forbidden to turn around, squat, or lie down, the important thing is to try never to leave. One of the two could lead, and the other could accept, follow the movement, and propose another. During the exercise, participants are guided to breathe slowly and deeply reflect on their experience. After the exercise, participants discuss their feelings.
